# Supplementary material for: APOE2 is associated with longevity independent of Alzheimer’s disease
Source: eLife. 2020 Oct 19;9:e62199. doi: 10.7554/eLife.62199 (PMC7588231; doi:10.7554/eLife.62199)
Supplement: Supplementary file 1. — (a) Subject characteristics in the overall NACC cohort. The sample median (minimum, maximum) is given for continuous variables. Information was unavailable regarding hypertension (N = 28), transient ischemic attack (N = 116), pacemaker (N = 260), angioplasty/endarterectomy/stent (N = 19), heart attack/cardiac arrest (N = 42), cardiac bypass procedure (N = 9), atrial fibrillation (N = 48), hypercholesterolemia (N = 102), congestive heart failure (N = 25), and stroke (N = 45). (b) Subject characteristics for individuals in the NACC cohort with a neuropathological assessment. The sample median (minimum, maximum) is given for continuous variables. Information was unavailable regarding hypertension (N = 3), transient ischemic attack (N = 24), pacemaker (N = 1), heart attack/cardiac arrest (N = 4), atrial fibrillation (N = 3), hypercholesterolemia (N = 17), congestive heart failure (N = 4), stroke (N = 7),. density of neocortical neuritic plaques CERAD score (N = 12), density of diffuse plaques CERAD score (N = 328), Braak NFT stage (N = 55), presence of vascular pathology (N = 51), and minimal amyloid pathology. (c) Association between APOE genotype and survival using the NACC data. HR = hazard ratio; CI = confidence interval; AD = Alzheimer’s disease; CV = cardiovascular. HRs, 95% CIs, and p-values result from Cox proportional hazards regression models. 1 These analyses were only performed when considering all subjects. 2 Cognitive status at final visit and AD at final visit were adjusted for only in the model involving all subjects. 3 CV factors included hypertension, transient ischemic attack, pacemaker, angioplasty/endarterectomy/stent, heart attack/cardiac arrest, atrial fibrillation, hypercholesterolemia, congestive heart failure, and stroke. 4 For the neuropathological assessment cohort, all models were additionally adjusted for CERAD diffuse plaque score, CERAD neuritic plaque score, Braak NFT stage, and the presence of vascular pathology. p-values<0.025 are cons [file elife-62199-supp1.docx]

**Supplementary File 1a.**

| Variable | All subjects (N=24,661) | *APOE3* (ε3/ε3) subjects (N=12,387) | *APOE2* (ε2/ε2 or ε2/ε3) subjects (N=2,269) | *APOE4* (ε3/ε4 or ε4/ε4) subjects (N=9,354) | ε2/ε4 subjects (excluded, N=651) |
| --- | --- | --- | --- | --- | --- |
| Age at initial visit (years) | 73 (18, 109) | 73 (18, 109) | 73 (20, 102) | 72 (20, 99) | 73 (22, 100) |
| Age at final visit (years) | 76 (18, 110) | 76 (18, 110) | 77 (20, 105) | 75 (20, 106) | 76 (22, 103) |
| Sex (female) | 13,882 (56.3%) | 7,016 (56.6%) | 1,294 (57.0%) | 5,185 (55.4%) | 387 (59.4%) |
| Race |  |  |  |  |  |
| White | 20,753 (84.2%) | 10,552 (85.2%) | 1,845 (81.3%) | 7,854 (84.0%) | 502 (77.1%) |
| Black | 2,887 (11.7%) | 1,221 (9.9%) | 343 (15.1%) | 1,187 (12.7%) | 136 (20.9%) |
| Other | 1,021 (4.1%) | 614 (5.0%) | 81 (3.6%) | 313 (3.3%) | 13 (2.0%) |
| Cognitive status at final visit |  |  |  |  |  |
| Normal cognition | 8,869 (36.0%) | 5,187 (41.9%) | 1,085 (47.8%) | 2,369 (25.3%) | 228 (35.0%) |
| Impaired but not MCI | 1,026 (4.2%) | 598 (4.8%) | 126 (5.6%) | 277 (3.0%) | 25 (3.8%) |
| MCI | 3,853 (15.6%) | 2,009 (16.2%) | 384 (16.9%) | 1,349 (14.4%) | 111 (17.1%) |
| Dementia | 10,913 (44.3%) | 4,593 (37.1%) | 674 (29.7%) | 5,359 (57.3%) | 287 (44.1%) |
| Clinically diagnosed AD at final visit | 8,704 (35.3%) | 3,325 (26.8%) | 449 (19.8%) | 4,685 (50.1%) | 245 (37.6%) |
| CV risk factors (present at any visit) | 76(18, 110) | 76 (18, 110) | 77 (20, 105) | 75 (20, 106) | 76 (22, 103) |
| Hypertension | 14,164 (57.5%) | 7,182 (58.0%) | 1,368 (60.4%) | 5,234 (56.0%) | 380 (58.4%) |
| Transient ischemic attack | 1,729 (7.0%) | 898 (7.3%) | 174 (7.7%) | 610 (6.6%) | 47 (7.3%) |
| Pacemaker | 1,103 (4.5%) | 552 (4.5%) | 121 (5.4%) | 406 (4.4%) | 24 (3.8%) |
| Angioplasty/ endarterectomy/stent | 2,071 (8.4%) | 1,064 (8.6%) | 187 (8.2%) | 764 (8.2%) | 56 (8.6%) |
| Heart attack/cardiac arrest | 1,798 (7.3%) | 939 (7.6%) | 173 (7.6%) | 651 (7.0%) | 35 (5.4%) |
| Cardiac bypass procedure | 1,260 (5.1%) | 658 (5.3%) | 104 (4.6%) | 472 (5.0%) | 26 (4.0%) |
| Atrial fibrillation | 2,494 (10.1%) | 1,331 (10.8%) | 247 (10.9%) | 851 (9.1%) | 65 (10.0%) |
| Hypercholesterolemia | 14,443 (58.8%) | 7,215 (58.5%) | 1,000 (44.3%) | 5,919 (63.5%) | 309 (47.5%) |
| Congestive heart failure | 1,021 (4.1%) | 569 (4.6%) | 127 (5.6%) | 302 (3.2%) | 23 (3.5%) |
| Stroke | 1,707 (6.9%) | 851 (6.9%) | 218 (9.6%) | 592 (6.3%) | 46 (7.1%) |
| Death | 5,413 (21.9%) | 2,624 (21.2%) | 444 (19.6%) | 2,196 (23.5%) | 149 (22.9%) |
| With Neuropathological assessment (%Death) | 3,528 (65.2%) | 1,700 (64.8%) | 282 (63.5%) | 1,452 (66.1%) | 94 (63.1%) |

**Supplementary File 1b.**

| Variable | All subjects (N=3,528) | *APOE3* (ε3/ε3) subjects (N=1,700) | *APOE2* (ε2/ε2 or ε2/ε3) subjects (N=282) | *APOE4* (ε3/ε4 or ε4/ε4) subjects (N=1,452) | ε2/ε4 subjects (N=94) |
| --- | --- | --- | --- | --- | --- |
| Age at initial visit (years) | 78 (26, 109) | 80 (26, 109) | 80 (38, 102) | 76 (31, 99) | 80 (49, 100) |
| Age at final visit (years) | 81 (26, 110) | 83 (26, 110) | 83 (38, 105) | 79 (31, 105) | 83 (52, 101) |
| Sex (female) | 1617 (45.8%) | 795 (46.8%) | 136 (48.2%) | 636 (43.8%) | 50 (53.2%) |
| Race |  |  |  |  |  |
| White | 3362 (95.3%) | 1634 (96.1%) | 269 (95.4%) | 1368 (94.2%) | 91 (96.8%) |
| Black | 120 (3.4%) | 38 (2.2%) | 11 (3.9%) | 69 (4.8%) | 2 (2.1%) |
| Other | 46 (1.3%) | 28 (1.6%) | 2 (0.7%) | 15 (1.0%) | 1 (1.1%) |
| Cognitive status at final visit |  |  |  |  |  |
| Normal cognition | 406 (11.5%) | 269 (15.8%) | 64 (22.7%) | 66 (4.5%) | 7 (7.4%) |
| Impaired but not MCI | 52 (1.5%) | 30 (1.8%) | 6 (2.1%) | 15 (1.0%) | 1 (1.1%) |
| MCI | 306 (8.7%) | 183 (10.8%) | 42 (14.9%) | 71 (4.9%) | 10 (10.6%) |
| Dementia | 2764 (78.3%) | 1218 (71.6%) | 170 (60.3%) | 1300 (89.5%) | 76 (80.9%) |
| Clinically diagnosed AD at final visit | 2046 (58.0%) | 824 (48.5%) | 101 (35.8%) | 1057 (72.8%) | 64 (68.1%) |
| CV risk factors (present at any visit) |  |  |  |  |  |
| Hypertension | 2044 (58.0%) | 1027 (60.4%) | 179 (63.5%) | 787 (54.3%) | 51 (54.3%) |
| Transient ischemic attack | 349 (10.0%) | 178 (10.5%) | 32 (11.4%) | 127 (8.8%) | 12 (12.9%) |
| Pacemaker | 260 (7.4%) | 140 (8.2%) | 27 (9.6%) | 89 (6.1%) | 4 (4.3%) |
| Angioplasty/ endarterectomy/stent | 367 (10.4%) | 178 (10.5%) | 28 (9.9%) | 150 (10.3%) | 11 (11.7%) |
| Heart attack/cardiac arrest | 393 (11.2%) | 214 (12.6%) | 28 (9.9%) | 144 (9.9%) | 7 (7.4%) |
| Cardiac bypass procedure | 264 (7.5%) | 139 (8.2%) | 20 (7.1%) | 100 (6.9%) | 5 (5.3%) |
| Atrial fibrillation | 549 (15.6%) | 288 (17.0%) | 41 (14.5%) | 205 (14.1%) | 15 (16.0%) |
| Hypercholesterolemia | 1879 (53.5%) | 899 (53.1%) | 115 (40.9%) | 829 (57.5%) | 36 (38.3%) |
| Congestive heart failure | 322 (9.1%) | 189 (11.1%) | 38 (13.5%) | 89 (6.1%) | 6 (6.4%) |
| Stroke | 401 (11.4%) | 203 (12.0%) | 48 (17.0%) | 141 (9.7%) | 9 (9.7%) |
| Density of neocortical neuritic plaques CERAD score |  |  |  |  |  |
| No neuritic plaques | 771 (21.9%) | 520 (30.7%) | 138 (49.1%) | 100 (6.9%) | 13 (13.8%) |
| Sparse neuritic plaques | 480 (13.7%) | 238 (14.0%) | 50 (17.8%) | 177 (12.2%) | 15 (16.0%) |
| Moderate neuritic plaques | 660 (18.8%) | 350 (20.7%) | 39 (13.9%) | 250 (17.3%) | 21 (22.3%) |
| Frequent neuritic plaques | 1605 (45.6%) | 586 (34.6%) | 54 (19.2%) | 920 (63.6%) | 45 (47.9%) |
| Density of diffuse plaques CERAD score |  |  |  |  |  |
| No diffuse plaques | 513 (16.0%) | 346 (22.6%) | 96 (37.5%) | 64 (4.8%) | 7 (8.0%) |
| Sparse diffuse plaques | 457 (14.3%) | 251 (16.4%) | 50 (19.5%) | 144 (10.8%) | 12 (13.8%) |
| Moderate diffuse plaques | 525 (16.4%) | 253 (16.5%) | 40 (15.6%) | 225 (16.9%) | 7 (8.0%) |
| Frequent diffuse plaques | 1705 (53.3%) | 679 (44.4%) | 70 (27.3%) | 895 (67.4%) | 61 (70.1%) |
| Braak NFT stage |  |  |  |  |  |
| 0 | 196 (5.6%) | 134 (8.0%) | 32 (11.5%) | 28 (1.9%) | 2 (2.1%) |
| I | 301 (8.6%) | 178 (10.6%) | 51 (18.3%) | 69 (4.8%) | 3 (3.2%) |
| II | 406 (11.6%) | 266 (15.8%) | 49 (17.6%) | 78 (5.4%) | 13 (13.8%) |
| III | 324 (9.3%) | 172 (10.2%) | 44 (15.8%) | 98 (6.8%) | 10 (10.6%) |
| IV | 494 (14.1%) | 246 (14.6%) | 47 (16.8%) | 183 (12.7%) | 18 (19.1%) |
| V | 639 (18.3%) | 254 (15.1%) | 21 (7.5%) | 341 (23.6%) | 23 (24.5%) |
| VI | 1113 (31.8%) | 413 (24.6%) | 34 (12.2%) | 641 (44.4%) | 25 (26.6%) |
| Vascular pathology | 3384 (97.3%) | 1601 (96.2%) | 265 (96.4%) | 1425 (98.8%) | 93 (98.9%) |
| Minimal amyloid pathology | 592 (16.8%) | 409 (24.1%) | 117 (41.6%) | 58 (4.0%) | 8 (8.5%) |

**Supplementary File 1c.**

|  | N | Survival at age 90, % (95% CI) | Adjusting for sex and race | | Adjusting for sex, race, cognitive status at final visit, and AD at final visit^1^ | | Adjusting for sex, race, cognitive status at final visit, AD at final visit^2^, and CV factors^3^ | |
| --- | --- | --- | --- | --- | --- | --- | --- | --- |
| ***APOE* genotype** |  |  | HR (95% CI) | P-value | HR (95% CI) | P-value | HR (95% CI) | P-value |
| All subjects (N=24,661) |  |  |  |  |  |  |  |  |
| *APOE3* (ε3/ε3) | 12,387 | 57.2 (55.5, 59.0) | 1.00 (reference) | N/A | 1.00 (reference) | N/A | 1.00 (reference) | N/A |
| *APOE2* (ε2/ε2 or ε2/ε3) | 2,269 | 64.8 (61.2, 68.7) | 0.84 (0.76, 0.93) | 0.0005 | 0.87 (0.78, 0.96) | 0.005 | 0.88 (0.80, 0.98) | 0.014 |
| *APOE4* (ε3/ε4 or ε4/ε4) | 9,354 | 40.2 (38.0, 42.5) | 1.52 (1.44, 1.61) | <0.0001 | 1.36 (1.28, 1.44) | <0.0001 | 1.34 (1.27, 1.43) | <0.0001 |
| ε2/ε4 | 651 | 43.5 (36.0, 52.5) | 1.24 (1.05, 1.46) | 0.010 | 1.15 (0.98, 1.36) | 0.091 | 1.12 (0.95, 1.33) | 0.17 |
| No AD at final visit (15,957) |  |  |  |  |  |  |  |  |
| *APOE3* (ε3/ε3) | 9,062 | 63.3 (61.1, 65.5) | 1.00 (reference) | N/A | N/A | N/A | 1.00 (reference) | N/A |
| *APOE2* (ε2/ε2 or ε2/ε3) | 1,820 | 67.5 (63.1, 72.2) | 0.88 (0.77, 1.00) | 0.052 | N/A | N/A | 0.86 (0.76, 0.98) | 0.028 |
| *APOE4* (ε3/ε4 or ε4/ε4) | 4,669 | 56.2 (52.3, 60.3) | 1.24 (1.13, 1.37) | <0.0001 | N/A | N/A | 1.26 (1.15, 1.39) | <0.0001 |
| ε2/ε4 | 406 | 56.9 (44.8, 72.2) | 1.03 (0.78, 1.36) | 0.83 | N/A | N/A | 1.03 (0.78, 1.36) | 0.84 |
| AD at final visit (N=8,704) |  |  |  |  |  |  |  |  |
| *APOE3* (ε3/ε3) | 3,325 | 49.4 (46.9, 52.0) | 1.00 (reference) | N/A | N/A | N/A | 1.00 (reference) | N/A |
| *APOE2* (ε2/ε2 or ε2/ε3) | 449 | 58.3 (52.2, 65.2) | 0.84 (0.71, 0.99) | 0.033 | N/A | N/A | 0.84 (0.71, 0.99) | 0.036 |
| *APOE4* (ε3/ε4 or ε4/ε4) | 4,685 | 33.2 (30.7, 35.8) | 1.47 (1.36, 1.59) | <0.0001 | N/A | N/A | 1.43 (1.33, 1.53) | <0.0001 |
| ε2/ε4 | 245 | 34.8 (26.4, 46.1) | 1.28 (1.04, 1.57) | 0.022 | N/A | N/A | 1.24 (1.00, 1.53) | 0.050 |
| Neuropathological assessment (N=3528)^4^ |  |  |  |  |  |  |  |  |
| *APOE3* (ε3/ε3) | 1700 | 24.1 (22.2, 26.2) | 1.00 (reference) | N/A | N/A | N/A | 1.00 (reference) | N/A |
| *APOE2* (ε2/ε2 or ε2/ε3) | 282 | 31.9 (2.9, 37.9) | 0.83 (0.72, 0.95) | 0.006 | N/A | N/A | 0.88 (0.76, 1.01) | 0.062 |
| *APOE4* (ε3/ε4 or ε4/ε4) | 1452 | 11.2 (9.7, 12.9) | 1.46 (1.34, 1.58) | <0.0001 | N/A | N/A | 1.41 (1.30, 1.53) | <0.0001 |
| ε2/ε4 | 94 | 20.2 (13.5, 20.2) | 1.06 (0.85, 1.32) | 0.60 | N/A | N/A | 0.90 (0.72, 1.12) | 0.33 |
| Minimal amyloid pathology (N=592) |  |  |  |  |  |  |  |  |
| *APOE3* (ε3/ε3) | 409 | 19.6 (16.1, 23.8) | 1.00 (reference) | N/A | N/A | N/A | 1.00 (reference) | N/A |
| *APOE2* (ε2/ε2 or ε2/ε3) | 117 | 28.2 (21.1, 37.7) | 0.83 (0.73, 0.94) | 0.003 | N/A | N/A | 0.78 (0.63, 0.96) | 0.021 |
| *APOE4* (ε3/ε4 or ε4/ε4) | 58 | 13.8 (7.2, 26.2) | 1.52 (1.41, 1.63) | <0.0001 | N/A | N/A | 1.36 (1.03, 1.82) | 0.033 |
| ε2/ε4 | 8 | 25.0 (7.5, 83.0) | 1.01 (0.82, 1.25) | 0.90 | N/A | N/A | 1.02 (0.50, 2.09) | 0.95 |

**Supplementary File 1d.**

|  | **ApoE2-TR** | **ApoE3-TR** | **ApoE4-TR** | ***Apoe*-KO** | ***p*-value** |
| --- | --- | --- | --- | --- | --- |
| **Survival cohort** | | | | | |
| **Total No.** | 29 | 27 | 34 | 28 | — |
| **Female No. (%)** | 17 (58.6) | 14 (51.9) | 20 (58.8) | 14 (50.0) | 0.8625 |
| **Age at behavior-tested at old age, month** | 23.5 ± 1.4 [21.1-24.6] | 23.8 ± 0.3 [23.3-24.1] | 24.1 ± 0.1 [23.9-24.3] | 22.5 ± 0.9 [21.8-23.9] | <0.0001 |
| **Median age at death, day [95%lower, higher]** | 911 [780-949] | 825 [783-853] | 752.5 [696-796] | 737.5 [660-817] | 0.0041 |
| **Found dead No. (%)** | 14 (48.3) | 19 (70.3) | 24 (70.6) | 21 (75.0) | 0.1413 |
| **Euthanized No. (%)** | 15 (51.7) | 8 (29.6) | 10 (29.4) | 7 (25.0) |  |
| **Hunched, weight loss, lethergic (% of euthanized)** | 3 | 6 | 5 | 4 | 0.0653 |
| **Tumor (% of euthanized)** | 2 | 2 | 3 | 0 | 0.2549 |
| **Recurrent UD, skin problem (% of euthanized)** | 5 | 0 | 2 | 2 | 0.3086 |
| **Prolapse (% of euthanized)** | 1 | 0 | 0 | 0 | 0.6448 |
| **Multiple (% of euthanized)** | 2 | 0 | 0 | 1 | 0.4415 |
| **Other reasons (due to end of study)** | 2 | 0 | 0 | 0 | 0.3323 |
| **Biochemically assessed cohort** | | | | | |
| **Young cohort No.** | 28 | 26 | 26 | 23 | — |
| **Female No. (%)** | 14 (50.0) | 12 (46.2) | 14 (53.8) | 11 (47.8) | 0.9520 |
| **Age at sacrifice, month** | 7.3 ± 0.7 [6.3-7.9] | 7.6 ± 0.2 [7.4-7.8] | 7.2 ± 0.3 [6.6-7.5] | 6.2 ± 0.8 [5.2-7.4] | <0.0001 |
| **Male body weight, gram** | 28.7 ± 2.1 [25.9-33.1] | 29.5 ± 2.7 [22.2-33.4] | 30.5 ± 2.8 [25.3-34.9] | 29.2 ± 2.4 [23.4-32.6] | 0.3432 |
| **Female body weight, gram** | 24.7 ± 2.5 [21.7-28.9] | 21.9 ± 1.9 [19.4-24.7] | 23.5 ± 1.7 [21.2-27.1] | 24.0 ± 2.6 [19.0-28.2] | 0.0190 |
| **Old cohort No.** | 27 | 17 | 16 | 17 | — |
| **Female No. (%)** | 10 (37.0) | 8 (47.1) | 10 (62.5) | 6 (35.3) | 0.3424 |
| **Age at sacrifice, month** | 22.8 ± 1.0 [21.3-24.7] | 22.6 ± 1.0 [21.7-24.5] | 22.4 ± 0.2 [22.2-22.7] | 22.8 ± 0.6 [21.7-24.1] | 0.3565 |
| **Male body weight, gram** | 31.6 ± 2.3 [27-36.9] | 33.5 ± 3.4 [28.8-38.4] | 32.7 ± 2.1 [30.4-35.8] | 29.5 ± 3.4 [20.2-32.8] | 0.0263 |
| **Female body weight, gram** | 25.1 ± 3.4 [19.3-30.0] | 25.2 ± 3.5 [22.6-33.7] | 25.4 ± 3.7 [17.8-29.3] | 26.2 ± 3.2 [22-30.3] | 0.9425 |

**Supplementary File 1e****.**

|  |  |  |  | *APOE2* vs. *APOE3* (reference) | | *APOE4* vs. *APOE3* (reference) | |
| --- | --- | --- | --- | --- | --- | --- | --- |
| **GDS item** | *APOE2* (ε2/ε2 or ε2/ε3, N=1,197) | *APOE3* (ε3/ε3, N=5,717) | *APOE4* (ε3/ε4 or ε4/ε4, N=2,616) | OR (95% CI) | P-value | OR (95% CI) | P-value |
| **Primary item** |  |  |  |  |  |  |  |
|  |  |  |  |  |  |  |  |
| Dropped many of your activities and interests | 149 (12.4%) | 830 (14.5%) | 349 (13.4%) | 0.80 (0.66, 0.96) | 0.019 | 0.98 (0.86, 1.13) | 0.82 |
|  |  |  |  |  |  |  |  |
| **Secondary items** |  |  |  |  |  |  |  |
|  |  |  |  |  |  |  |  |
| Satisfied with your life | 1102 (92.1%) | 5190 (90.9%) | 2379 (91.0%) | 1.19 (0.95, 1.49) | 0.14 | 1.00 (0.85, 1.18) | 0.96 |
| Feel that your life is empty | 57 (4.8%) | 310 (5.4%) | 139 (5.3%) | 0.85 (0.64, 1.14) | 0.29 | 1.03 (0.83, 1.27) | 0.80 |
| Often get bored | 93 (7.8%) | 497 (8.7%) | 196 (7.5%) | 0.87 (0.69, 1.09) | 0.22 | 0.84 (0.71, 1.00) | 0.053 |
| In good spirits most of the time | 1134 (94.7%) | 5410 (94.7%) | 2479 (94.8%) | 0.99 (0.75, 1.30) | 0.91 | 1.02 (0.83, 1.26) | 0.85 |
| Afraid something bad happen is going to happen to you | 80 (6.7%) | 437 (7.6%) | 211 (8.1%) | 0.90 (0.70, 1.15) | 0.38 | 1.08 (0.91, 1.28) | 0.39 |
| Feel happy most of the time | 1099 (91.9%) | 5206 (91.3%) | 2388 (91.3%) | 1.07 (0.86, 1.35) | 0.54 | 0.99 (0.84, 1.16) | 0.87 |
| Often feel helpless | 72 (6.0%) | 471 (8.2%) | 180 (6.9%) | 0.69 (0.53, 0.89) | 0.005 | 0.90 (0.75, 1.07) | 0.24 |
| Prefer to stay at home rather than going out and doing new things | 274 (22.9%) | 1376 (24.1%) | 583 (22.3%) | 0.94 (0.81, 1.09) | 0.39 | 0.94 (0.84, 1.05) | 0.24 |
| Feel that you have more problems with memory than most | 126 (10.5%) | 710 (12.4%) | 378 (14.5%) | 0.86 (0.70, 1.05) | 0.13 | 1.18 (1.03, 1.35) | 0.020 |
| Think it is wonderful to be alive now | 1134 (94.7%) | 5433 (95.1%) | 2494 (95.4%) | 0.93 (0.70, 1.24) | 0.62 | 0.93 (0.74, 1.16) | 0.52 |
| Feel pretty worthless the way you are now | 52 (4.3%) | 272 (4.8%) | 91 (3.5%) | 0.88 (0.65, 1.19) | 0.40 | 0.82 (0.64, 1.05) | 0.12 |
| Feel full of energy | 755 (63.1%) | 3717 (65.0%) | 1786 (68.3%) | 0.95 (0.83, 1.08) | 0.40 | 1.07 (0.97, 1.18) | 0.19 |
| Feel that your situation is hopeless | 23 (1.9%) | 174 (3.0%) | 69 (2.6%) | 0.62 (0.40, 0.96) | 0.034 | 0.96 (0.72, 1.28) | 0.79 |
| Think that most people are better off than you are | 37 (3.1%) | 175 (3.1%) | 78 (3.0%) | 1.00 (0.70, 1.44) | 1.00 | 0.99 (0.75, 1.30) | 0.92 |
| Total GDS score | 1.44 (0, 2) | 1.55 (0, 2) | 1.46 (0, 2) | 0.92 (0.82, 1.04) | 0.17 | 0.99 (0.91, 1.07) | 0.73 |

**Supplementary File 1f.**

|  | **Travel distance** | | **Time mobile** | | **Rearing events** | |
| --- | --- | --- | --- | --- | --- | --- |
|  | ***r*** | ***p*-value** | ***r*** | ***p*-value** | ***r*** | ***p*-value** |
| **CX apoE** | 0.69 | <0.0001 | 0.59 | <0.0001 | 0.62 | <0.0001 |
| **HP apoE** | 0.68 | <0.0001 | 0.57 | <0.0001 | 0.56 | <0.0001 |
| **CSF apoE** | 0.57 | <0.0001 | 0.42 | 0.0043 | 0.47 | 0.006 |
| **Plasma apoE** | 0.64 | <0.0001 | 0.43 | 0.0018 | 0.54 | <0.0001 |
| **CX PSD95** | -0.15 | 1 | -0.19 | 1 | -0.29 | 0.1630 |
| **HP PSD95** | 0.27 | 0.2658 | 0.28 | 0.2519 | 0.15 | 1 |
| **CX GFAP** | 0.21 | 1 | 0.06 | 1 | -0.09 | 1 |
| **HP GFAP** | 0.14 | 1 | 0.10 | 1 | -0.10 | 1 |
| **CX CD11b** | 0.09 | 1 | 0.06 | 1 | -0.06 | 1 |
| **HP CD11b** | 0.15 | 1 | 0.27 | 0.3072 | -0.07 | 1 |
| **CX IL1β** | 0.24 | 0.5742 | 0.09 | 1 | 0.21 | 1 |
| **CX TNFα** | 0.09 | 1 | -0.07 | 1 | 0.06 | 1 |
| **Plasma CRP** | 0.09 | 1 | 0.137 | 1 | 0.07 | 1 |
| **Plasma total cholesterol** | -0.04 | 1 | -0.24 | 0.6085 | -0.04 | 1 |
| **Plasma HDL cholesterol** | 0.30 | 0.1747 | 0.31 | 0.1338 | 0.45 | 0.0013 |
| **Plasma non-HDL cholesterol** | -0.05 | 1 | -0.25 | 0.5500 | -0.04 | 1 |
| **Plasma triglyceride** | 0.50 | 0.0001 | 0.26 | 0.4152 | 0.46 | 0.0007 |

**Supplementary File 1g.**

| Contributors (PI) | NIA/NIH Funding Resource |
| --- | --- |
| Eric Reiman, MD | P30 AG019610 |
| Neil Kowall, MD | P30 AG013846 |
| Scott Small, MD | P50 AG008702 |
| Allan Levey, MD, PhD | P50 AG025688 |
| Andrew Saykin, PsyD | P30 AG010133 |
| Marilyn Albert, PhD | P50 AG005146 |
| Bradley Hyman, MD, PhD | P50 AG005134 |
| Ronald Petersen, MD, PhD | P50 AG016574 |
| Mary Sano, PhD | P50 AG005138 |
| Steven Ferris, PhD | P30 AG008051 |
| M. Marsel Mesulam, MD | P30 AG013854 |
| Jeffrey Kaye, MD | P30 AG008017 |
| David Bennett, MD | P30 AG010161 |
| Charles DeCarli, MD | P30 AG010129 |
| Frank LaFerla, PhD | P50 AG016573 |
| David Teplow, PhD | P50 AG016570 |
| Douglas Galasko, MD | P50 AG005131 |
| Bruce Miller, MD | P50 AG023501 |
| Russell Swerdlow, MD | P30 AG035982 |
| Linda Van Eldik, PhD | P30 AG028383 |
| John Trojanowski, MD, PhD | P30 AG010124 |
| Oscar Lopez, MD | P50 AG005133 |
| Helena Chui, MD | P50 AG005142 |
| Roger Rosenberg, MD | P30 AG012300 |
| Thomas Montine, MD, PhD | P50 AG005136 |
| Sanjay Asthana, MD, FRCP | P50 AG033514 |
| John Morris, MD | P50 AG005681 |
| Stephen Strittmatter, MD, PhD | P50 AG047270 |
